# Precision Care for Hereditary Urologic Cancers: Genetic Testing, Counseling, Surveillance, and Therapeutic Implications

**DOI:** 10.3390/curroncol32120698

**Published:** 2025-12-11

**Authors:** Takatoshi Somoto, Takanobu Utsumi, Rino Ikeda, Naoki Ishitsuka, Takahide Noro, Yuta Suzuki, Shota Iijima, Yuka Sugizaki, Ryo Oka, Takumi Endo, Naoto Kamiya, Hiroyoshi Suzuki

**Affiliations:** Department of Urology, Toho University Sakura Medical Center, Sakura 285-8741, Japan; takatoshi.soumoto@med.toho-u.ac.jp (T.S.); rino.ikeda@med.toho-u.ac.jp (R.I.); naoki.ishitsuka@med.toho-u.ac.jp (N.I.); takahide.noro@med.toho-u.ac.jp (T.N.); yuta.suzuki@med.toho-u.ac.jp (Y.S.); shouta.iijima@med.toho-u.ac.jp (S.I.); yuuka.kizuki@med.toho-u.ac.jp (Y.S.); ryou.oka@med.toho-u.ac.jp (R.O.); takumi.endou@med.toho-u.ac.jp (T.E.); naoto.kamiya@med.toho-u.ac.jp (N.K.); hiroyoshi.suzuki@med.toho-u.ac.jp (H.S.)

**Keywords:** hereditary cancer syndromes, genetic counseling, prostate cancer, renal cell carcinoma, upper tract urothelial carcinoma, pheochromocytoma and paraganglioma, adrenocortical carcinoma, Lynch syndrome

## Abstract

Hereditary predisposition substantially shapes prevention and management across urologic oncology. This narrative review synthesizes contemporary, practice-oriented guidance on whom to test, what to test, how to act on results, and how to implement care equitably for hereditary forms of prostate cancer, renal cell carcinoma (RCC), urothelial carcinoma, pheochromocytoma/paraganglioma (PPGL), and adrenocortical carcinoma (ACC). We delineate between forms of indication-driven germline testing (e.g., universal testing in metastatic prostate cancer; early-onset, bilateral/multifocal, or syndromic RCC; reflex tumor mismatch repair (MMR)/microsatellite instability (MSI) screening in upper-tract urothelial carcinoma (UTUC); universal testing in PPGL; universal *TP53* testing in ACC) and pair these strategies with minimum actionable gene sets and syndrome-specific surveillance frameworks. Key points include targeted prostate-specific antigen screening in *BRCA2* carriers and the impact of *BRCA/ATM* variants on reclassification during active surveillance; major hereditary RCC syndromes with genotype-tailored surveillance and pathway-directed therapy (e.g., HIF-2α inhibition for von Hippel–Lindau disease); UTUC/bladder cancer in Lynch syndrome with tumor MMR/MSI screening, annual urinalysis (selective cytology), and immunotherapy opportunities in deficient MMR disease/MSI-H; PPGL management emphasizing universal germline testing, intensified surveillance for *SDHB*, cortical-sparing adrenalectomy, and emerging HIF-2α inhibition; and ACC care modified by Li–Fraumeni syndrome (minimization of radiation/genotoxic therapy with whole-body imaging surveillance). Testicular germ cell tumor remains largely polygenic, with no routine germline testing in typical presentations. Finally, we provide pre-/post-test genetic-counseling checklists and mainstreamed workflows with equity metrics to operationalize precision care and close real-world access gaps.

## Simple Summary

Some urologic cancers arise from inherited genetic variation. This review offers pragmatic guidance on when to pursue genetic testing, which genes are most consequential, how results should direct screening and treatment, and how to ensure equitable access for all patients. We address prostate cancer, renal cell carcinoma, and urothelial carcinoma, adrenal tumors, and testicular germ cell tumors. We specify who should be offered testing and highlight genotype-informed therapies. We also provide counseling checklists and clinic workflows that health systems can adopt. Collectively, these recommendations can orient future studies, inform clinical and coverage policies, and advance equitable, real-world cancer care.

## 1. Introduction

Hereditary determinants are increasingly recognized across urologic oncology. Prostate cancer is among the most heritable common malignancies, with twin studies estimating ~57% heritability and demonstrating substantial genetic contributions across multiple tumor types [1]. Upper tract urothelial carcinoma (UTUC) functions as a sentinel malignancy for Lynch syndrome; accordingly, routine tumor assessment for mismatch repair (MMR) deficiency is warranted in many settings, given the nontrivial prevalence of MMR loss in UTUC and its association with Lynch syndrome [2]. By contrast, testicular germ cell tumors (TGCTs) exhibit pronounced familial aggregation but predominantly reflect a polygenic architecture rather than a single high-penetrance gene [3].

These inherited susceptibilities underpin the expanding role of germline testing and genetic counseling in urologic cancer care. Contemporary consensus recommends germline evaluation for all men with metastatic prostate cancer—and for selected men with high-risk localized disease or strong family histories—to inform early detection, cascade testing, and therapy selection [4]. In kidney cancer, recognition of hereditary renal cell carcinoma (RCC) syndromes and the meaningful yield of germline findings in advanced RCC support targeted testing strategies that consider age at onset, multifocal/bilateral disease, histology, and family history [5].

Germline findings increasingly guide management. In metastatic castration-resistant prostate cancer (mCRPC), pathogenic variants in homologous recombination repair genes—especially *BRCA2/BRCA1*—identify patients who benefit from PARP inhibition, improving progression-free outcomes over novel hormonal agents [6]. Similarly, identification of deficient MMR (dMMR)—whether due to Lynch syndrome or somatic inactivation—can predict sensitivity to programmed cell death-1 (PD-1) blockade across tumor types, a principle that extends to urothelial and prostate cancers with dMMR/microsatellite instability (MSI)-high biology [7,8].

**Figure 1:**
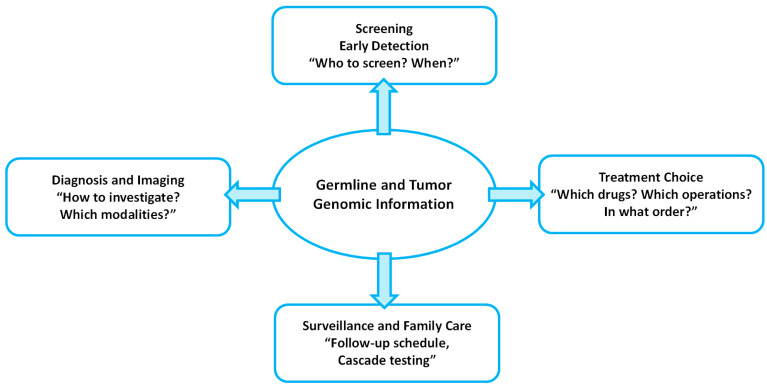
Conceptual framework illustrating how germline and tumor genomic findings shape urologic cancer care.

Taken together, these examples underscore that hereditary cancer syndromes determine not only who should undergo screening, but also how patients navigate the entire diagnostic and therapeutic pathway. Germline and tumor genomic findings now inform multiple decision points along the care continuum: (i) identifying clinical scenarios that should prompt genetic evaluation; (ii) selecting and sequencing diagnostic tests, including reflex tumor assays; (iii) optimizing imaging modality and intensity; (iv) individualizing the timing and extent of surgery; (v) choosing systemic therapies that exploit pathway-specific vulnerabilities; and (vi) designing long-term surveillance strategies and cascade testing for at-risk relatives. Figure 1 summarizes this germline-informed care pathway and the critical junctures at which genomic information intersects with screening, diagnosis, imaging, local therapy, systemic treatment, and survivorship across hereditary urologic cancers.

Germline and tumor genomic information functions as a central hub influencing four major domains along the care continuum: (1) screening and early detection (defining who should undergo screening and the appropriate age or timing to initiate it); (2) diagnosis and imaging (determining the extent of diagnostic workup and the selection of imaging modalities); (3) therapeutic decision-making (guiding the choice and sequencing of local and systemic treatments); and (4) surveillance and familial management (designing syndrome-specific follow-up protocols and implementing cascade genetic testing in at-risk relatives).

As these germline-informed pathways expand, ensuring equitable access becomes critical, with programs tracking offer and uptake, turnaround times, and cascade completion as core equity metrics. Despite clear clinical utility, disparities persist in access to testing and counseling. Real-world data show that a minority of eligible men complete germline testing, with lower uptake among historically underserved populations—differences that narrow in equal-access systems—highlighting system-level barriers and the need for mainstreamed, equitable pathways [9]. This review synthesizes current indications, counseling frameworks, syndrome-specific surveillance, and therapeutic implications for hereditary urologic cancers, while outlining practical steps to reduce access gaps.

This article is a narrative, practice-oriented review rather than a systematic review. We identified relevant literature through non-systematic searches of PubMed/MEDLINE and guideline repositories (e.g., NCCN, EAU, ESMO, ASCO, Endocrine Society, ESE/ENSAT) up to November 2025, restricted to English-language publications. We prioritised contemporary international and national guidelines, expert consensus statements, large cohort studies, pivotal clinical trials, and high-impact reviews, with targeted selection of key hereditary urologic cancer syndromes (prostate, RCC/RTPS, UTUC/bladder, PPGL, ACC, and TGCT). Reference lists of retrieved articles and major guidelines were also screened to capture additional relevant sources. Given the narrative design, we did not perform formal risk-of-bias assessment or meta-analysis.

## 2. Prostate Cancer

### 2.1. Genetic Predisposition and Testing Guidelines

Prostate cancer has a substantial hereditary component; twin and family studies estimate that ~57–60% of risk is attributable to inherited genetic factors [1]. Notably, early investigations in Ashkenazi Jewish hereditary prostate cancer pedigrees reported no enrichment of *BRCA1/2* founder mutations, underscoring population-specific genetic architectures [10]. Key high-risk germline susceptibility genes include *BRCA2*, *BRCA1*, DNA MMR genes, and the prostate-specific *HOXB13* variant, among others. *HOXB13* was the first gene conclusively linked to familial prostate cancer [11,12,13]. The *HOXB13* G84E variant confers an approximately 3–5-fold increase in overall prostate cancer risk, with even higher relative risk up to 8–10-fold for early-onset disease [14]. Penetrance is substantial; a population-based analysis reported upper-bound estimates approaching ~60% by age 80 in selected cohorts [14]. In light of these insights, contemporary consensus guidelines strongly endorse germline genetic testing for all men with metastatic prostate cancer, as well as for those with early high-risk disease or family histories suggestive of hereditary cancer syndromes. These recommendations are affirmed and extended by current guidelines; National Comprehensive Cancer Network (NCCN) explicitly endorses germline testing for all metastatic cases and for selected high-risk localized cases irrespective of family history [4,15]. Recommended gene panels prioritize DNA repair and Lynch syndrome genes—with *BRCA2*, *BRCA1*, and MMR genes as top targets in metastatic disease. In earlier-stage or screening contexts, *BRCA2* and *HOXB13* are highlighted for evaluation. Men harboring pathogenic *BRCA2* variants are now advised to initiate prostate cancer screening, prostate-specific antigen (PSA) and digital rectal examination, at younger ages, given elevated risk and the propensity for aggressive tumors [15,16]. Major oncology guidelines have converged on these points: all recommend germline genomic testing in men with advanced or very high-risk prostate cancer, regardless of family history, to identify actionable alterations such as DNA repair defects or MMR deficiency [15,16]. Germline mutations are detected in an estimated 12–17% of men with metastatic prostate cancer (vs. ~5% in localized disease), underscoring the clinical yield of testing in advanced cases [17,18]. Practical indications for germline testing in prostate cancer, together with key clinical implications, are summarized in Table 1.

### 2.2. Surveillance and Counseling for Carriers

Identification of pathogenic variants enables tailored screening and prevention. Men with pathogenic *BRCA2* or *BRCA1* variants are generally counseled to begin prostate cancer screening 5–10 years earlier than average, around age 40 [4,15]. Prospective data from the IMPACT study support targeted PSA-based screening in men with germline *BRCA1/2* variants: the initial screening round demonstrated a higher detection rate and enrichment for clinically significant disease in *BRCA2* carriers vs. non-carriers, and interim analyses continue to support annual PSA testing in *BRCA2* carriers aged 40–69 [19,20]. Expert groups also suggest considering intensified surveillance in carriers of other predisposition genes (e.g., *HOXB13*, *ATM*, *CHEK2*) or in those with strong family histories, although formal guidelines are still evolving [15,16]. U.S. screening recommendations now include men with high-risk germline mutations (such as *BRCA2* or Lynch syndrome MMR genes) or high-risk ancestry (e.g., African or Ashkenazi Jewish heritage) within the “high-risk” category for earlier PSA-based screening, typically in the 40s [15,16]. Such individuals—along with men from prostate cancer families—should engage in informed discussions about earlier screening given their elevated lifetime risk. Genetic counseling is recommended for all patients and families undergoing testing to aid interpretation and to facilitate cascade testing of at-risk relatives [15,16]. Among men already managed with active surveillance, germline *ATM* or *BRCA1/2* pathogenic variants have been associated with increased risk of grade reclassification, suggesting tighter monitoring schedules and careful selection for surveillance in such carriers [21]. Counseling should also address psychosocial considerations and implications for other associated cancers.

Beyond PSA-based algorithms, germline status also intersects with imaging-driven risk stratification. Systematic biopsy alone frequently underestimates pathologic Gleason grade at prostatectomy, and newer histologic metrics and imaging-based pathways have been developed to reduce misclassification [22]. Contemporary guidelines now endorse multiparametric magnetic resonance imaging (mpMRI) prior to biopsy and at the outset of active surveillance; mpMRI-targeted or combined targeted/systematic biopsy improves detection of clinically significant cancer while reducing overdiagnosis of low-grade disease [23,24]. In MRI-led surveillance cohorts, serial mpMRI interpreted using PRECISE criteria helps identify radiologic progression and can inform the timing of repeat biopsy and transition to definitive treatment [24]. For carriers of high-risk germline variants such as BRCA2 or ATM—who experience higher rates of upgrading during surveillance [21,24]—clinicians may reasonably adopt a lower threshold for MRI-directed re-biopsy or definitive treatment, or avoid active surveillance altogether when additional adverse clinicopathologic features are present [8,17,21,24]. Emerging data suggest that prostate-specific membrane antigen positron emission tomography/computed tomography (PSMA PET/CT) may further refine selection for, and monitoring of, active surveillance by revealing otherwise occult higher-grade lesions; early systematic reviews indicate that PSMA PET can upstage a subset of ostensibly low-risk tumors and may therefore reduce inappropriate surveillance [25]. In practice, PSMA PET is not yet routine for germline carriers, but may be considered in selected men with discordant clinical and MRI findings, particularly when a high-risk germline mutation raises concern for undersampling of aggressive disease [25,26].

### 2.3. Therapeutic Implications

Germline testing increasingly informs therapeutic decision-making. The paradigm is PARP inhibition in men with DNA repair gene alterations. Landmark trials demonstrate that patients with metastatic castration-resistant prostate cancer (mCRPC) harboring homologous recombination repair (HRR) gene mutations—particularly BRCA2 or BRCA1—derive substantial benefit from PARP inhibitors such as olaparib. In the phase III PROfound trial, olaparib more than doubled radiographic progression-free survival (rPFS) (median 7.4 vs. 3.6 months) compared with standard hormonal therapy in men with *BRCA1/2*- or *ATM*-mutated mCRPC, and improved overall survival (OS) (median ~19 vs. 15 months in the *BRCA1/2/ATM* cohort) despite crossover [6]. PARP-based first-line combinations have further expanded options: olaparib plus abiraterone (*BRCA*-mutated mCRPC) and talazoparib plus enzalutamide (HRR-mutated mCRPC) significantly improve rPFS and are incorporated into recent guidelines [27,28]. In PROpel, rPFS was significantly prolonged in the ITT population (HR 0.66, 95% CI 0.54–0.81), while regulatory review concluded that efficacy was primarily attributable to the BRCA-mutated subgroup (exploratory analyses: rPFS HR 0.24; OS HR 0.30) [27].

These data have practical consequences for treatment sequencing. In men with germline *BRCA2* or other HRR mutations, many clinicians now consider earlier use of PARP inhibitors—either as part of first-line combination regimens or soon after failure of androgen receptor signalling inhibitors (ARSIs)—rather than reserving PARP inhibition for late lines, and they deliberately weigh the timing of docetaxel, ARSIs, and PARP inhibitors to maintain efficacy within the window of maximal PARP sensitivity [6,25,27]. Germline and somatic testing are complementary in this context. Tumor genomic profiling can document biallelic inactivation of HRR genes or identify somatic-only HRR alterations in men who test negative on germline panels, thereby broadening eligibility for PARP inhibition and other targeted approaches [17,25,29]. Conversely, when a germline mutation is identified, confirming tumor-level involvement helps explain exceptional sensitivity (e.g., biallelic *BRCA2* loss) versus attenuated benefit when the wild-type allele is retained [6,17]. The presence of a germline HRR variant also influences management beyond the PARP setting; for example, some experts favor earlier intensification with docetaxel or potent ARSIs in *BRCA2* carriers with high-volume metastatic disease, given their higher risk of early progression on androgen-deprivation therapy alone [17,18,26,30].

In parallel, patients with MMR-deficient or MSI-high prostate cancers, which often related to Lynch syndrome, may benefit from immune checkpoint blockade. Pembrolizumab has shown durable activity in MMR-deficient solid tumors and carries an indication for any unresectable MSI-high tumor irrespective of site [7]. Thus, a germline diagnosis of Lynch syndrome can enable access to immunotherapy (e.g., pembrolizumab) in the rare MSI-high prostate cancers.

## 3. RCC and Renal Tumor Predisposition Syndrome (RTPS)

### 3.1. Overview of Hereditary RCC and RTPS

This chapter focuses on RCC and, where pertinent, RTPS—most notably tuberous sclerosis complex (TSC), in which angiomyolipoma (AML) represents a mesenchymal PEComa rather than RCC [31,32,33]. A meaningful subset of RCC arises from hereditary syndromes; contemporary estimates suggest that roughly 5–8% of cases are attributable to inherited pathogenic variants [31,32]. Recognition is critical, as hereditary RCC often presents at younger ages and with multifocal or bilateral tumors. Clinical features that should prompt genetic evaluation include early-onset RCC (typically <46 years), bilateral or multifocal tumors, uncommon histologies (non-clear-cell subtypes), and/or a positive family history of RCC [34,35]. Patients meeting these criteria should be referred for genetic counseling and multigene panel testing. Some guidelines and consensus further clarified that any patient with RCC diagnosed before age 46, or with ≥1 first- or second-degree relative with RCC, or with bilateral/multiple tumors, or with histology suggestive of a known syndrome should undergo germline risk assessment (Table 2) [34,35,36].

Hereditary renal neoplasia spans (i) epithelial RCC subtypes (e.g., clear cell, papillary, chromophobe, oncocytic/hybrid tumors) arising in classic RCC syndromes, and (ii) non-epithelial renal tumors encountered in predisposition syndromes (e.g., AML in TSC). Distinguishing these entities is essential for genetic counseling, surveillance intervals, and surgical thresholds. Recent series and reviews of TSC-associated RCC highlight earlier onset (mean ~30s), female predominance, and diverse histologies (chromophobe-like, hybrid oncocytic/chromophobe, eosinophilic variants), underscoring implications for diagnosis and nephron-sparing planning [35,36,37]. Consider germline testing in patients with early-onset disease, bilateral or multifocal renal tumors, non-clear-cell or hybrid oncocytic histologies, a personal/family history of syndrome-defining extra-renal tumors [36,37].

### 3.2. Major Hereditary RCC Syndromes and TSC

The principal autosomal dominant syndromes account for most hereditary RCC: von Hippel–Lindau (VHL), Hereditary Leiomyomatosis and RCC (HLRCC), Hereditary Papillary RCC (HPRC), Birt–Hogg–Dubé (BHD), and BAP1 tumor predisposition syndrome (BAP1-TPDS). Each is defined by a distinct causative gene and renal tumor phenotype:

VHL—caused by germline VHL mutations. Predisposes primarily to clear-cell RCC, often with multiple bilateral tumors. Reported lifetime RCC risk ranges ~24–45% [32,38], with some cohorts indicating even higher penetrance (up to ~70% by later age) [32,38]. Classic VHL also features central nervous system hemangioblastomas, retinal angiomas, pheochromocytomas, pancreatic neuroendocrine tumors, and various cystic lesions.

HLRCC—caused by FH mutations. Produces an aggressive type 2 papillary RCC (often solitary, high grade) with early metastatic potential. RCC risk is estimated at ~20–30% by age 70 [39,40], although precise risk estimation remains challenging (one analysis assumed ~21% by age 70). Hallmark extrarenal findings include multiple cutaneous leiomyomas and, in women, early and numerous uterine fibroids.

HPRC—caused by activating MET proto-oncogene mutations. Confers a near-100% lifetime risk of papillary RCC (type 1), typically bilateral and multifocal, usually arising in mid-adulthood. Unlike other syndromes, HPRC is generally limited to the kidneys, with no systemic manifestations [31,32].

BHD—caused by FLCN mutations. Yields a spectrum of renal tumor histologies; hybrid oncocytic/chromophobe tumors are characteristic, though clear-cell or papillary RCC may also occur. Renal cancer risk is ~15–30% [41]. Distinctive features include fibrofolliculomas (benign follicular tumors on the face/neck) and pulmonary cysts with risk of spontaneous pneumothorax.

BAP1-TPDS—germline BAP1 mutations confer an elevated risk of clear-cell RCC—albeit less frequently—alongside uveal melanoma and mesothelioma; 2023 European guidance outlines surveillance for carriers [42].

TSC—caused by pathogenic variants in TSC1/TSC2. Renal disease is dominated by AMLs and cysts. RCC risk is increased but substantially lower than in other classic hereditary RCC syndromes (e.g., VHL, HLRCC, BHD), with available series suggesting single-digit to low double-digit percentage risks over a lifetime [33,37]. Extra-renal features include neurocutaneous manifestations and, in women, lymphangioleiomyomatosis (LAM) [33,43].

### 3.3. Surveillance Strategies for Mutation Carriers

Once a hereditary RCC syndrome is identified, proactive surveillance for renal tumors is essential. However, the intensity and timing of imaging should be tailored to the tumor biology of each syndrome rather than applying a uniform template [31,32,34,35]. Differences in growth kinetics and metastatic potential among VHL disease, HLRCC, HPRC, and BHD syndrome underlie the distinct surveillance intervals and surgical thresholds recommended for each condition [31,32,34,35,38,40,41].

In VHL, clear-cell RCCs typically grow slowly over years, yet carriers remain at lifelong risk for multifocal, bilateral tumors; natural history series and expert guidelines indicate that metastatic events are uncommon below approximately 3 cm, supporting a strategy of active surveillance with nephron-sparing intervention when the largest solid lesion approaches 3 cm to balance oncologic control and renal preservation [31,32,38,44]. Accordingly, VHL surveillance guidelines recommend annual renal MRI or CT beginning in childhood or adolescence, integrated within broader multi-organ surveillance that includes periodic brain and spine MRI, ophthalmologic examinations, and plasma or urine metanephrines for pheochromocytoma [38,44]. Because surveillance in VHL typically begins in childhood and continues lifelong, structured transition from pediatric to adult care is essential.

BHD shares an indolent renal course in many cases: FLCN-associated tumors—often hybrid oncocytic/chromophobe or chromophobe-like lesions—tend to grow slowly and can be monitored until the largest solid component reaches approximately 3 cm, at which point nephron-sparing surgery or ablation is recommended [31,32,41]. A practical approach is to obtain baseline abdominal MRI in early adulthood (around age 20 years) with repeat imaging every 1–3 years, with intervals shortened if lesions are present or demonstrate growth [41].

By contrast, HLRCC is driven by biallelic inactivation of FH, leading to fumarate accumulation, pseudohypoxia, and a highly aggressive type 2 papillary RCC phenotype [39,40]. FH-deficient tumors may metastasize when very small, and case series report advanced disease arising from lesions well below the standard 3 cm threshold [39,40]. For this reason, consensus statements recommend starting renal MRI in early adolescence (often as young as 8–10 years) and repeating annually throughout life [40]. Any solid or complex renal lesion in a confirmed FH-mutation carrier should prompt urgent assessment in an experienced center, with a low threshold for early, wide-margin excision even at subcentimeter to small-centimeter sizes, rather than the deferred intervention strategy used for VHL or BHD [40]. This “fast-track” approach reflects biology: FH-deficient RCC is typically high grade, shows early nodal or distant spread, and carries a markedly worse prognosis than VHL-, BHD-, or MET-associated tumors if not resected promptly [39,40]. Given the risk of RCC and uterine fibroids in adolescence and early adulthood, care should anticipate transition from pediatric/Adolescents and Young Adults (AYA) services to adult multidisciplinary clinics.

HPRC, caused by activating MET proto-oncogene variants, occupies an intermediate position [31,35]. MET-driven type 1 papillary RCCs are usually bilateral and multifocal, but individual lesions often behave more indolently than FH-deficient cancers and rarely metastasize when small [31,35]. Ultrasound performs poorly for papillary histology, so contrast-enhanced MRI is preferred for surveillance, generally starting around age 30 years (earlier in families with very young-onset disease) at 1–2-year intervals [31,32,35]. Together, these examples illustrate that the “3 cm rule” is appropriate for relatively indolent VHL-, BHD-, and HPRC-associated lesions, but unsafe for FH-deficient RCC, where biologic aggressiveness mandates earlier imaging and lower surgical thresholds [31,32,35,40,41].

For BAP1-TPDS, expert-consensus recommendations advise annual renal MRI beginning at approximately age 30 (ultrasound may be used when MRI is unavailable), with shortened intervals when lesions are detected. Given the extra-renal phenotype, programs should be embedded in multidisciplinary care with annual dermatologic and ophthalmologic examinations for cutaneous and ocular manifestations [42].

For TSC surveillance and AML-first strategy, consensus statements recommend lifelong renal MRI every 1–3 years from diagnosis (intervals shortened with growth), with at least annual blood pressure and kidney function monitoring; clinicians should document baseline AML burden and growth kinetics and screen for extra-renal disease, notably pulmonary LAM in women, within a multidisciplinary program [33,45]. Intervention for large or enlarging AMLs (commonly ≥3–4 cm, aneurysmal change, or symptoms) aims to prevent hemorrhage and preserve parenchyma, using mTOR inhibition (e.g., everolimus) or selective embolization; partial nephrectomy/ablation is reserved for refractory cases or suspected malignancy [33,45]. In TSC, many renal and extra-renal manifestations arise in childhood or adolescence, and surveillance should be coordinated across pediatric and adult services with clear transition plans.

Across all of these syndromes, extra-renal surveillance is not optional but integral to care, because non-renal manifestations often precede or parallel kidney tumors. VHL programs must incorporate regular retinal examinations, brain and spine MRI, and biochemical testing for pheochromocytoma alongside renal imaging [38,44]. HLRCC surveillance requires close collaboration with dermatology and gynecology for cutaneous leiomyomas and early, symptomatic uterine fibroids, which frequently drive presentation [40]. BHD mandates baseline and, when indicated, follow-up chest CT and counseling about pneumothorax risk and triggers (e.g., diving, unpressurized air travel), in addition to renal imaging [41]. BAP1-TPDS calls for annual dermatologic and ophthalmologic assessment and awareness of mesothelioma risk [42].

TSC care spans neurology, pulmonology, nephrology, and dermatology, with structured evaluation for epilepsy, neurocognitive issues, pulmonary LAM, and cutaneous lesions as outlined in consensus statements [33]. Multidisciplinary clinics that integrate genetics, urology, nephrology, dermatology, neurology, ophthalmology, gynecology, and pulmonology—as appropriate to the specific syndrome—and that adhere to structured protocols such as those summarized in Table 3 offer the best opportunity to detect both renal and extra-renal manifestations early and to coordinate timing of interventions.

### 3.4. Treatment Considerations

Recognition of a hereditary syndrome materially influences RCC management. Surgical strategies are tailored to syndrome-specific tumor biology—prioritizing nephron-sparing approaches and staged interventions in view of multifocality and recurrence [34,35,36]. Patients with hereditary RCC and AML commonly undergo serial partial nephrectomies or ablative procedures, intervening once lesions exceed defined size thresholds while observing smaller foci to defer intervention (Table 2).

In recent years, systemic therapies directed at pathogenic pathways have emerged for hereditary RCC. A paradigm shift is the HIF-2α inhibitor belzutifan (MK-6482) for VHL-associated RCC. In a phase II trial of VHL patients with multiple clear-cell RCCs managed nonoperatively, belzutifan achieved a 49% objective response rate in renal tumors, with disease control in essentially all patients; at 2 years, 96% remained progression-free on therapy [46]. Responses were durable, and the agent also reduced the size of VHL-related lesions in other organs (e.g., pancreatic NETs, hemangioblastomas) [46]. Thus, HIF-2α inhibition offers an effective systemic option that can defer or diminish the need for repeated surgeries in VHL disease [47]. Targeted therapies are being explored across other syndromes as well. MET tyrosine kinase inhibitors, such as savolitinib, have shown activity in MET-driven papillary RCC, including hereditary forms [48]. Similarly, RCCs in HLRCC, which is marked by dysregulated metabolism (fumarate accumulation) and HIF stabilization, have demonstrated sensitivity to metabolic strategies and VEGF/EGFR blockade. An NIH phase II study of bevacizumab (VEGF antibody) plus erlotinib (EGFR inhibitor) reported a 72% confirmed response rate in advanced HLRCC-associated kidney cancers [49], with median rPFS of 21 months, indicating substantial activity in this aggressive subtype. Immune checkpoint inhibitors are now standards of care in sporadic advanced RCC and are also employed in metastatic hereditary RCC, although syndrome-specific trials are lacking [36].

## 4. Urothelial Carcinoma (UC)

### 4.1. UTUC

UTUC has a well-established association with Lynch syndrome. UTUC is the third most common malignancy in Lynch syndrome (after colorectal and endometrial cancers) [50,51,52], particularly linked to germline *MSH2* and *MSH6* mutations. Given this strong association, current guidelines advocate heightened vigilance for Lynch syndrome in any patient presenting with UTUC. Some guidelines, for example, recommend that all patients diagnosed with UTUC at age ≤ 60 undergo evaluation for Lynch syndrome (e.g., tumor MMR immunohistochemistry and/or germline testing) [50,53]. In practice, many centers perform MMR protein IHC or microsatellite instability testing on UTUC specimens as a screening step—often regardless of age—because of the relatively high Lynch prevalence. Indeed, reliance solely on the “under-60” criterion risks missed cases, as Lynch syndrome-associated UTUC can arise in the 60s or 70s; some experts increasingly consider universal MMR/MSI screening of all UTUC tumors, mirroring colorectal protocols [50,53]. Notably, one series found that 93% of Lynch-related UTUCs among confirmed carriers exhibited loss of an MMR protein by IHC [2]. The experts also advise genetic counseling and testing when a UTUC patient has a personal or family history suggestive of Lynch-spectrum cancers [50,53].

For confirmed Lynch syndrome mutation carriers, surveillance for urothelial malignancies is an important—albeit debated—component of care. Since 2021, many experts recommend at least annual urinalysis (and consider urine cytology in *MSH2* carriers), coupled with education on hematuria and a low threshold for evaluation [51,54,55]. The goal is early detection of asymptomatic hematuria that may signal an upper tract tumor. Some specialized clinics go further—recommending urine cytology and even periodic ureteroscopy or upper-tract imaging in select high-risk individuals—though these strategies lack proven mortality benefit and are not universally endorsed. In practice, most clinicians at least perform annual urinalysis and counsel Lynch carriers regarding UTUC symptoms (flank pain, hematuria) [51,55]. If a Lynch carrier has a history of ureteral or renal pelvis cancer, intensive contralateral surveillance is warranted given the elevated risk of second urothelial primaries. Preventively, smoking cessation is essential—as in sporadic disease—since smoking synergistically heightens urothelial cancer risk in this population. Beyond routine urinalysis and cytology, a growing armamentarium of urine-based biomarkers is being evaluated as adjuncts to cystoscopy and imaging in urothelial cancer surveillance. Several FDA-approved assays (e.g., NMP22, BTA, UroVysion FISH) and newer multiplex panels such as Cxbladder have demonstrated higher sensitivity than cytology alone for detecting recurrent high-grade bladder cancer, albeit with trade-offs in specificity and cost [56]. More recently, epigenetic tests that interrogate DNA methylation signatures in voided urine—exemplified by the Bladder EpiCheck assay, which analyzes methylation at a panel of loci—have shown promising performance in non-muscle-invasive bladder cancer follow-up, offering high negative predictive values and improving detection of high-grade recurrences when combined with cytology and cystoscopy [57,58]. Data in Lynch-associated UTUC are limited, and none of these assays is currently guideline-mandated for hereditary syndromes; nevertheless, in specialized centers, urine-based biomarkers may be considered as complementary tools to triage hematuria or to refine surveillance intensity in very high-risk individuals, while preserving cystoscopy and upper-tract imaging as the backbone of follow-up [51,53,59].

Management of UTUC in Lynch syndrome follows standard oncologic principles (endoscopic resection or nephroureterectomy for localized disease; platinum-based chemotherapy for advanced disease), with the added consideration that these tumors frequently exhibit high microsatellite instability [50,53]. MMR-deficient UTUCs (and bladder cancers) are typically sensitive to immune checkpoint inhibition. Advanced or metastatic UTUC in a Lynch syndrome patient can be treated with anti–PD-1/PD-L1 therapy—such as pembrolizumab or nivolumab—analogous to MSI-high colorectal cancer [50,53]. Pembrolizumab holds a tissue-agnostic approval for unresectable MSI-H solid tumors, and case reports document dramatic responses in MMR-deficient UC [7]. Another key consideration is the high risk of synchronous or metachronous malignancies: a substantial fraction of Lynch patients with UTUC will develop a second cancer (e.g., colorectal carcinoma, another UTUC, or bladder cancer). Therefore, long-term follow-up and age-appropriate screening for other Lynch-associated neoplasms (e.g., colonoscopy) are critical [50,53].

From a hereditary perspective, upper-tract and bladder urothelial tumors do not behave identically and should not be managed under a single urothelial “template”. In Lynch syndrome, UTUC is both more strongly enriched and more likely to represent the sentinel malignancy than bladder cancer, particularly in *MSH2* carriers [2,51,55,59]. Cohort and registry studies demonstrate that lifetime UTUC risks in *MSH2* carriers can exceed those for bladder cancer, with a high rate of metachronous contralateral upper-tract tumors and frequent multifocal disease [51,59]. Consequently, surveillance paradigms for Lynch carriers have traditionally prioritized early detection of asymptomatic upper-tract disease through annual urinalysis (±cytology in *MSH2* carriers), prompt investigation of any hematuria, and a lower threshold for repeat upper-tract imaging after an index UTUC [51,60]. Bladder cancers, by contrast, are less commonly Lynch-related, typically arise later in life, and are more strongly influenced by acquired risk factors such as smoking; their cumulative risk, while elevated over the general population, remains lower than that of UTUC in most Lynch series [51,55,55,59,60]. In practice, bladder tumors in Lynch carriers are often detected incidentally through the same urine-based surveillance used for UTUC or when hematuria triggers cystoscopy. These distinctions underscore why guidelines emphasize UTUC as a sentinel cancer warranting systematic surveillance, whereas “hereditary bladder cancer” per se lacks dedicated protocols beyond Lynch-directed assessment and symptom-driven evaluation [51,54,59,60,61].

### 4.2. Bladder Cancer

Although purely hereditary bladder cancer is uncommon, genetics can be contributory in specific contexts. Unlike prostate, kidney, and UTUC, no single high-penetrance gene is known to frequently cause bladder cancer in the general population. Nevertheless, Lynch syndrome modestly increases bladder cancer risk; recent reviews estimate ~12% cumulative risk by age 70 in male *MSH2* carriers—higher than population norms [55,59]. Lynch-associated bladder cancers are usually MMR-deficient; thus, as with UTUC, any urothelial tumor in a known Lynch patient warrants MMR analysis, and eligible patients with advanced disease may receive immunotherapy [55,59]. Beyond Lynch syndrome, familial clustering of bladder cancer has long been observed, but disentangling heritable risk from shared environmental exposures (notably tobacco and occupational carcinogens) is challenging. Routine germline testing is not recommended for bladder cancer absent indications for a Lynch work-up or suspicion of a broader hereditary syndrome based on personal/family history [55,59].

Accordingly, genetic evaluation in bladder cancer typically centers on assessing for Lynch syndrome when high-risk features are present—for instance, MMR deficiency on pathology, or a family history enriched for colon, endometrial, or other Lynch-spectrum cancers. It is noteworthy that UTUC are far more commonly Lynch-related than bladder tumors [55,59]. There are no dedicated surveillance protocols for “hereditary bladder cancer.” Lynch carriers may undergo annual urine testing for hematuria, which could also flag bladder lesions, yet there is no evidence-based mandate for prophylactic cystoscopy or imaging solely for bladder cancer in Lynch syndrome [55,59,62]. In practice, annual urinalysis performed for UTUC surveillance simultaneously raises suspicion for bladder cancers. Any Lynch carrier with unexplained hematuria should receive prompt evaluation (including cystoscopy and upper-tract imaging). Table 4 summarizes the surveillance focus for Lynch-associated upper tract versus bladder urothelial carcinoma.

## 5. Pheochromocytoma and Paraganglioma (PPGL)

PPGLs are rare neuroendocrine neoplasms arising from the adrenal medulla (pheochromocytoma) or extra-adrenal paraganglia (paraganglioma). They cause secondary hypertension and catecholamine crises, with presentations spanning adolescence through late adulthood. Features suggestive of heritability include bilateral adrenal disease, multifocal/extra-adrenal tumors, and early onset. Contemporary care requires multidisciplinary coordination (endocrinology, surgery, oncology, nuclear medicine) and lifelong follow-up [63,64]. Surveillance in MEN2 and VHL carriers often begins in childhood, and timing of adrenal and thyroid surgery must be discussed within families as part of pediatric endocrine and surgical planning.

PPGLs have one of the highest hereditary fractions among solid tumors; approximately 30–40% harbor pathogenic germline variants across two principal biological clusters: pseudohypoxia (*SDHx*, VHL, *EPAS1*, *FH*) and kinase signaling (*RET*, *NF1*, *TMEM127*, *MAX*) [65]. Given this high germline diagnostic yield (approximately 30–40%)—and because ~10–15% of apparently sporadic cases still carry a causative variant—international guidelines and expert consensus recommend genetic counselling and multigene germline testing for all PPGL patients, rather than a selective approach [66]. Mutation status shapes phenotype and risk: SDHB carriers have the highest metastatic propensity, whereas multiple endocrine neoplasia type 2 (MEN) (*RET*) and VHL frequently present with bilateral adrenal tumors [65]. Identification of a germline variant also enables cascade testing and surveillance in relatives [66].

Carriers require annual biochemical screening (plasma-free or 24-h urinary fractionated metanephrines) plus periodic whole-body imaging, tailored to genotype [64,66]. A pragmatic approach includes skull-base-to-pelvis MRI every 2–3 years, intensified for *SDHB* (given higher metastatic risk) and initiated in childhood for MEN2 and VHL [64,66]. Structured surveillance programs detect smaller, localized tumors and reduce PPGL-specific morbidity among *SDHB* carriers [64,66].

Beyond surveillance, germline-defined clusters also inform imaging and treatment. Pseudohypoxia-related tumors (cluster 1; SDHx, VHL, EPAS1, FH) usually show strong uptake on somatostatin receptor PET/CT (±^18^F-FDG PET), whereas MIBG avidity is variable; recent reviews therefore favor somatostatin receptor PET/CT as the most sensitive whole-body functional study in SDHx-related disease, reserving ^123^I/^131^I-MIBG for clearly MIBG-avid lesions or when SSTR imaging is unavailable [65,66,67]. By contrast, kinase signaling–related tumors (cluster 2; RET, NF1, TMEM127, MAX) often remain strongly MIBG-avid and can be staged effectively with ^123^I-MIBG in addition to CT/MRI [65,66]. Clinically, this means that in MEN2 and VHL, cortical-sparing adrenalectomy is reasonable in carefully selected bilateral cases, whereas in SDHB-associated PPGL the higher metastatic risk generally favors total adrenalectomy or wide resection and, when systemic treatment is required, early consideration of peptide receptor radionuclide therapy (PRRT) or ^131^I-MIBG rather than repeated external-beam radiotherapy or alkylating chemotherapy [65,66,67,68].

In hereditary bilateral disease (e.g., VHL, MEN2), cortical-sparing adrenalectomy can control tumors while avoiding lifelong adrenal insufficiency. A multicenter registry of 625 patients with bilateral pheochromocytomas showed no survival decrement with cortical-sparing surgery and substantially lower steroid dependence compared with bilateral total adrenalectomy [64,66]. Belzutifan (HIF-2α inhibitor) became the first oral HIF-2α–targeted therapy approved in the U.S. for locally advanced, unresectable, or metastatic PPGL, supported by LITESPARK-015 (ORR ~26%; durable responses and reductions in antihypertensive dosing) [67,68,69].

## 6. Adrenocortical Carcinoma (ACC)

ACC is a rare, aggressive malignancy with bimodal age distribution and frequent cortisol/androgen excess. R0 resection is pivotal, yet recurrence is common; standardized surveillance and evidence-based adjuvant care are essential [70]. A meaningful subset is hereditary—predominantly Li–Fraumeni syndrome (LFS, germline TP53)—and many patients lack classic family histories. Accordingly, contemporary ESE/ENSAT and GENTURIS guidelines, supported by expert consensus, recommend universal germline TP53 testing for ACC, with genetic counselling and cascade testing for relatives, with particularly strong evidence in pediatric and adolescent cases [71].

LFS follows the Toronto protocol (clinical assessment/abdominal ultrasound every 6 months in childhood plus annual whole-body and brain MRI, continued into adulthood) [71]. After ACC resection, typical follow-up is chest/abdominal cross-sectional imaging every 3–4 months for 2 years, then every 6–12 months until year 5, with hormone monitoring tailored to functional status [70,71]. In LFS, germline TP53 status therefore not only justifies minimizing radiotherapy and alkylating chemotherapy when feasible, favoring surgery and mitotane due to heightened second-primary risks, but also supports MRI-based whole-body surveillance with selective use of ^18^F-FDG PET/CT at key decision points and early referral to high-volume centers for open en bloc adrenalectomy [70,71]. For pediatric ACC, TP53 status also informs family screening, timing of imaging, and survivorship planning during transitions from pediatric to adult oncology and endocrinology.

## 7. Testicular Germ Cell Tumor (TGCT)

TGCT is the most common solid tumor in young men and remains highly curable (>95% overall survival) with modern multimodal care. Standard therapy is high orchiectomy followed by risk-adapted surveillance, adjuvant chemotherapy, or retroperitoneal lymph node dissection according to histology and stage [72]. Unlike other urologic cancers, high-penetrance monogenic syndromes are rare. Familial clustering exists (4–8× risk in first-degree relatives), but epidemiology and GWAS support a polygenic architecture; type II TGCT derives from germ cell neoplasia in situ and is highly chemosensitive, underpinning outcomes [72]. Routine germline panel testing is not indicated in typical presentations. Large genome-wide association studies have identified dozens of common risk loci for TGCT, each conferring modest relative risks, supporting a primarily polygenic etiology rather than a single high-penetrance gene [3,72]. Rarely, TGCT may arise in the context of broader syndromes (e.g., Klinefelter syndrome, certain sex chromosome or testis development disorders), or in families with multiple very early-onset primaries. In such settings—multiple primaries, strong familial clustering at young ages, or syndromic presentations—referral for genetics evaluation may be considered, typically in a research context or via specialized germ cell tumor clinics rather than through routine panel testing. When patients with familial TGCT ask about genetic testing, clinicians should explain that, at present, no clinically validated germline panel or polygenic risk score is recommended for routine care, and that management continues to rely on standard orchiectomy, risk-adapted surveillance, and timely cisplatin-based therapy.

Klinefelter syndrome (KS) (47, XXY) is disproportionately linked to mediastinal (extragonadal) non-seminomatous GCT in adolescents/young adults; ~3% of male pediatric/AYA GCTs occur in KS—testing is reasonable in males presenting with mediastinal GCT [73]. For men with TGCT, standard practice includes offering sperm banking before cisplatin-based chemotherapy or radiotherapy, particularly in those with bilateral or metachronous tumors [72].

## 8. Genetic Testing and Counseling in Hereditary Urologic Cancers

Genetic counseling helps patients and families understand hereditary cancer risk and make informed decisions about testing, surveillance, and risk-reducing interventions (Table 5) [74,75,76]. In urologic oncology, counseling should accompany any guideline-indicated germline test request and be framed as a longitudinal process rather than a single pre-test conversation. Contemporary models emphasize structured pre- and post-test discussions, explicit explanation of possible results (pathogenic/likely pathogenic variants, variants of uncertain significance (VUS), and negative or indeterminate findings) and their implications for relatives, and integration of these steps into multidisciplinary care pathways and electronic health records. The framework below summarizes recommendations and practical implementation.

### 8.1. Overview of Germline Testing Technologies and Test Selection

Targeted multigene next-generation sequencing (NGS) panels, whole-exome sequencing (WES), and whole-genome sequencing (WGS) are the main germline testing modalities for hereditary cancer. Panels interrogate a predefined set of susceptibility genes (typically tens to a few hundred) at high depth and with relatively straightforward interpretation, and they remain the workhorse of clinical germline testing [77,78]. WES surveys most coding exons genome-wide but has more variable coverage and generates a larger set of variants to interpret, whereas WGS captures both coding and non-coding regions and, with appropriate bioinformatics, can detect single-nucleotide variants, small insertions/deletions, copy-number variants (CNVs), and structural rearrangements in a single assay [79,80]. CNVs are also assayed by targeted methods such as multiplex ligation-dependent probe amplification (MLPA), which is widely used for BRCA1/2 and mismatch-repair genes [81], and increasingly by depth-of-coverage algorithms applied to panel or exome data, with good concordance to MLPA when pipelines are validated [82,83].

In hereditary urologic oncology, indication-based targeted panels are generally preferred as first-line tests because they offer a practical balance between diagnostic yield, turnaround time, and interpretability [84,85,86]. These panels usually include DNA damage-repair genes (e.g., BRCA1, BRCA2, ATM, CHEK2, PALB2), mismatch-repair genes (Lynch syndrome), and syndrome-specific genes such as VHL, FH, FLCN, and MET [84,85,86]. WES or WGS is typically reserved for patients with a strong but unexplained hereditary phenotype (very early onset, marked familial clustering, atypical tumor spectra, or negative panel testing despite high clinical suspicion) or for research and national genome programs [77,79,87,88]. In these settings, genome-wide approaches can increase diagnostic yield and allow re-analysis as new genes are discovered, but they are more costly, require specialised bioinformatics and robust frameworks for managing incidental and secondary findings, and are therefore currently regarded as complementary rather than routine first-line tests in hereditary urologic cancers [77,80,89].

### 8.2. Pre-Test Counseling Principles

Indications and test selection. Confirm that germline testing is warranted based on personal/family history or tumor features. Choose an appropriately scoped assay—from single-gene tests (VHL, *FH*, *TP53*) to multigene panels covering HRR, MMR, and other relevant loci—according to the clinical context. Integrate somatic tumor profiling when it can guide therapy or refine germline risk (e.g., *BRCA* in prostate tumor or deficient MMR in UTUC should trigger confirmatory germline testing and targeted therapies).

Pre-test counseling and consent. Educate patients on purpose/scope (diagnostic vs. prognostic), possible results (pathogenic variant, variant of uncertain significance (VUS), negative) and their implications for both the individual and at-risk relative, and the plan for result-contingent management or periodic reanalysis. Clarify that patients may decline any or all components of testing and that their decision will not compromise standard oncologic care. Discuss potential downstream consequences for life and disability insurance, employment, and reproductive planning, and, where applicable, outline legal protections against genetic discrimination [74,75,76]. Explain how and when cascade testing for relatives would be offered if a syndrome is confirmed, including age-appropriate timing and options for surveillance or prevention. Obtain informed consent that addresses data use and sharing, possible secondary or incidental findings, and responsibilities for recontact if variant classifications change; review specimen logistics, turnaround time, and coordination with tumor genomic testing. Genetic counselling should also address psychosocial and practical issues, including anxiety, guilt, family communication about hereditary risk, and potential implications for life and disability insurance. Wherever possible, referral to psychosocial oncology services and provision of plain-language family letters or resources can facilitate informed cascade testing and support shared decision-making within families [74,75,76].

### 8.3. Post-Test Counseling and Result-Specific Management

Management should be individualized to the result and to the family context. For pathogenic/likely pathogenic (P/LP) variants, provide a written action plan covering (1) surveillance schedules, including organ-specific imaging and laboratory monitoring; (2) therapy implications, such as eligibility for targeted agents, intensity of systemic therapy, and avoidance of genotoxic regimens in certain syndromes; and (3) cascade testing with age- and organ-specific guidance for relatives. Encourage sharing of family letters and coordinate testing through genetics services or urology-led mainstream pathways [74,75,76]. For VUS, emphasize that a hereditary syndrome has not been confirmed, that clinical management should remain driven by personal and family history rather than the variant itself, and that the classification may change over time as evidence accumulates. Document the VUS, register it with the testing laboratory, and explain how patients will be notified of reclassification, encouraging re-contact if they move or change providers [75]. For negative results, distinguish clearly between a “true negative” (where a known familial P/LP variant has been excluded) and an uninformative negative (no variant found in the context of suggestive history). In the former case, reassure patients that their risk approximates that of the general population for that syndrome; in the latter, discuss residual uncertainty, panel limitations, and whether tumor sequencing or future expanded testing might be informative if clinical suspicion remains high. Across all result types, provide psychosocial support, patient-facing summaries, and any required documentation or referrals, and revisit testing options as new therapies or family history information emerge [75].

### 8.4. Implementation Strategies for Mainstreaming and Equity

Mainstreaming improves uptake and turnaround while reserving genetics specialists for complex cases [90]. Successful mainstream programs in breast, ovarian, pancreatic, and prostate cancer have shown that concise training of non-genetic healthcare professionals, standardized consent materials, and close collaboration with familial cancer centers can maintain the quality of genetic care while scaling access [91,92]. Use EMR order sets/templates to standardize counseling content. Tele-genetics (phone/video) broadens access and reduces travel/time burdens; ensure options for patients with limited technology [93]. Track equity metrics (offer/uptake, time-to-result, cascade completion) and address gaps; nationwide data show demographic/insurance disparities requiring system-level fixes [94], consistent with oncology cohort findings [95].

Dedicated hereditary cancer programs illustrate how these principles can be operationalized in practice. The IMPACT and related targeted prostate cancer screening studies integrate germline BRCA1/2 and mismatch repair variants with tailored PSA screening protocols, structured pre- and post-test counseling, and systematic cascade testing in at-risk men across multiple countries, demonstrating long-term engagement and earlier detection in high-risk carriers [19,20]. Similarly, VHL Alliance–accredited clinical care centers and familial RCC/VHL programs at major academic institutions deliver protocolized imaging surveillance, coordinated surgical timing, and family-based counseling through multidisciplinary clinics spanning urology, medical oncology, genetics, neurosurgery, and ophthalmology [38,44]. These models provide a blueprint for urologic oncology services seeking to embed hereditary cancer care into routine workflows while preserving specialist oversight.

In practical terms, an implementation package for hereditary urologic cancers can include: EMR prompts to flag “red flag” clinical scenarios; standardized pre-test consent templates; streamlined ordering pathways with embedded panel choices; integration of tele-genetics for pre- and post-test counselling; clearly defined roles for nurse navigators in coordinating testing and cascade communication; and standard operating procedures for contacting at-risk relatives. Core equity metrics include the proportion of eligible patients offered testing, uptake among those offered, turnaround time from ordering to result disclosure, and completion rates of cascade testing in first-degree relatives. When gaps are identified—for example, lower uptake in specific demographic or insurance groups—targeted interventions such as navigation support, translated materials, community outreach, and feedback to referring clinicians should be considered. In lower-resource settings, a stepwise approach focusing on careful family history, red-flag identification, and limited but high-yield panels may be more feasible than comprehensive genomic testing, whereas high-resource centers can implement fully mainstreamed multi-gene and WGS-enabled programs [91,92,93,94,95].

## 9. Limitations and Future Directions

This review is narrative rather than systematic and is based on targeted searches of guidelines, key trials, and high-impact studies; as such, some relevant publications may not have been captured, and we did not perform formal risk-of-bias assessment or meta-analysis. Recommendations also reflect current international and national guidelines, which vary across regions and are rapidly evolving as new genes, assays, and therapies enter practice. For many hereditary syndromes—particularly rarer entities such as HLRCC, BAP1-TPDS, PPGL, ACC, and TGCT—evidence for surveillance intervals, imaging modalities, and systemic treatment strategies comes from small cohorts, retrospective series, or expert consensus rather than prospective, syndrome-specific trials.

Future work should address these gaps through prospective natural history studies, pragmatic trials of surveillance strategies, and evaluation of pathway-directed therapies in hereditary cohorts. Cost-effectiveness analyses and implementation research are needed to clarify how best to deploy germline and tumor testing in diverse health systems, including resource-limited settings. Finally, coordinated hereditary cancer programs and registries across urologic oncology will be critical to refine risk estimates, optimize resource allocation, and ensure that advances in genomics translate into improved and equitable outcomes for patients and their families.

## 10. Conclusions

Hereditary predisposition affects all major urologic cancers more than was previously appreciated. Across prostate cancer, hereditary RCC syndromes, Lynch-associated urothelial carcinoma, PPGL, and ACC, germline and tumor genomic findings now inform not only who should be tested, but also how we screen, stage, operate, treat, and follow patients, while testicular germ cell tumor remains largely a polygenic outlier.

In practice, the priorities are straightforward: use guideline-based, indication-driven germline testing; translate pathogenic results into concrete changes in screening, imaging, surgical planning, systemic therapy, and cascade testing; and avoid overreacting to VUS or uninformative negatives. Embedding streamlined testing and counseling pathways into routine urologic care, supported by multidisciplinary teams, is essential to deliver these benefits equitably. Future work should clarify the role of broader sequencing (including WGS), refine risk prediction with clinical and genomic data, and build coordinated hereditary cancer programs and registries so that genomic information consistently translates into earlier detection, more rational therapy, and better outcomes for patients and their families.

## Figures and Tables

**Table 1:** Indications for Germline Testing and Key Implications in Hereditary Prostate Cancer.

Clinical Scenario/Syndrome	Key Genes (Typical Panel)	Candidates for Testing	Key Management Implications
Metastatic prostate cancer (any histology)	HRR genes (BRCA2, BRCA1, ATM, CHEK2, PALB2, RAD51D, etc.)	All men with metastatic hormone-sensitive or castration-resistant prostate cancer	Enables PARP inhibitor use and intensified systemic therapy; prompts cascade testing.
High-/very-high–risk or node-positive localized prostate cancer; strong family history	HRR genes ± HOXB13	Men with high-/very-high–risk or node-positive disease; men ≤ 60 years or with multiple affected first-degree relatives	Supports tailored PSA screening and lower biopsy threshold in carriers; informs use or avoidance of conservative active surveillance.
Prostate cancer with features suggestive of Lynch syndrome	MMR genes (MLH1, MSH2, MSH6, PMS2, EPCAM)	Men with prostate cancer plus personal or family history suggestive of Lynch syndrome	Confirms need for colonoscopic and UTUC surveillance; may identify candidates for PD-1 blockade in dMMR/MSI-high disease.
Unaffected men from high-risk families	Same panel as proband	Adult male first-degree relatives in families with a known pathogenic variant	Enables predictive testing; supports earlier and risk-adapted PSA-based screening.

HRR, homologous recombination repair; MMR, mismatch repair; UTUC, upper-tract urothelial carcinoma; PSA, prostate-specific antigen; dMMR, deficient mismatch repair; MSI, microsatellite instability.

**Table 2:** Indications for Germline Risk Assessment in Patients with RCC [34,35].

Trigger Category	Operational Criterion
Age at diagnosis	RCC < 46 years
Tumor number/laterality	Bilateral and/or multifocal RCC
Family history	≥1 first- or second-degree relative with RCC
Histology suggestive of a syndrome	Non-clear-cell or hybrid oncocytic patterns
Syndromic/extra-renal features	Findings typical of a hereditary syndrome
Tumor pathology/genomics	IHC or sequencing indicating a germline pathway
Clinician judgment	Early onset with atypical features or multiple triggers

IHC: immunohistochemistry, RCC: renal cell carcinoma.

**Table 3:** Carrier Surveillance Intervals and Modalities.

Syndrome (Gene)	Renal Imaging	Extra-Renal Screening	Intervention Threshold	Special Cautions
VHL (VHL)	Abdominal MRI every 12 months	Ophthalmology Brain/spine MRI Plasma/urine metanephrines	3 cm (nephron-sparing)	Multidisciplinary protocol
HLRCC (*FH*)	Kidney MRI every 12 months from early adolescence (some start at 8–10 years)	Gynecology (uterine); Dermatology (cutaneous)	Early surgery, even <3 cm	Low threshold; rapid referral
HPRC (*MET*)	Kidney MRI every 12–24 months from ~age 30 (shorten if growth) Ultrasound not preferred	—	3 cm (nephron-sparing)	Anticipate staged procedures
BHD (*FLCN*)	Kidney MRI every 1–2 years from ~age 20 Ultrasound if MRI unavailable	Baseline chest CT Pneumothorax risk education	3 cm → nephron-sparing/ablation	Avoid diving/high-pressure exposure
BAP1-TPDS (*BAP1*)	Kidney MRI every 12 months from ~age 30 (shorten if lesions) Ultrasound if MRI unavailable	Dermatology (skin) Ophthalmology (uveal) Mesothelioma awareness	Lower threshold for intervention on enhancing solid mass; prioritize nephron-sparing	Aggressive biology; asbestos avoidance; coordinate care
TSC (*TSC1/2*)	Kidney MRI every 1–3 years lifelong Annual blood pressure and eGFR	Neurology Dermatology Screen LAM (women)	AML ≥ 3–4 cm or rapid growth → intervene	Hemorrhage risk; coordinate care

AML: angiomyolipoma, BHD: Birt–Hogg–Dubé, CT: computed tomography, eGFR: estimated glomerular filtration rate, FH: fumarate hydratase, FLCN: folliculin, HLRCC: hereditary leiomyomatosis and renal cell carcinoma, HPRC: hereditary papillary renal carcinoma, LAM: lymphangioleiomyomatosis, MET: MET proto-oncogene (hepatocyte growth factor receptor), MRI: magnetic resonance imaging, TSC: tuberous sclerosis complex, VHL: von Hippel–Lindau.

**Table 4:** Surveillance focus for Lynch-associated upper-tract vs. bladder urothelial carcinoma.

Context in Lynch Syndrome	Predominant Genes	Target Population	Surveillance Focus
UTUC	Mainly *MSH2* (also *MLH1*, *MSH6*, *PMS2*)	Lynch syndrome carriers from ~30–35 years; particularly MSH2 carriers; patients with prior UTUC	Annual urinalysis (±cytology in *MSH2* carriers); prompt CT/MR urography for any hematuria; after nephroureterectomy, contralateral upper-tract imaging at least annually for several years.
Bladder cancer	Mainly *MSH2*	Lynch syndrome carriers, especially older men with *MSH2* variants	Urine-based surveillance for UTUC usually detects many bladder tumors; cystoscopy triggered by hematuria, atypical cytology, or imaging findings; once diagnosed, follow standard NMIBC/MIBC guidelines.
Lynch syndrome without known UC	MMR genes (*MLH1*, *MSH2*, *MSH6*, *PMS2*, *EPCAM*)	All confirmed Lynch syndrome carriers	Counsel regarding UTUC as a sentinel cancer; yearly urinalysis; low threshold to investigate hematuria; coordinate with colorectal and gynecologic surveillance according to Lynch syndrome guidelines.

UC, urothelial carcinoma; UTUC, upper-tract urothelial carcinoma; MMR, mismatch repair; NMIBC, non-muscle-invasive bladder cancer; MIBC, muscle-invasive bladder cancer.

**Table 5:** Pre- and Post-Test Genetic Counseling Checklist.

Domain	Clinician Notes (What to Cover)	Patient-Facing Phrasing (Plain Language)
Purpose & scope	Germline vs. tumor testing, panel, lab, turnaround time, possible extra sample	“This looks for inherited and tumor changes to guide your care and your family’s.”
Results & reclassification	P/LP, VUS, negative; lab reclassification policy	“Results may show a risk, be uncertain, or show none. We’ll update you if meanings change.”
Family impact (cascade)	Who to offer testing; logistics; letters for relatives	“If an inherited change is found, close relatives can get a simple test.”
Privacy, documentation & coverage	Data handling, EHR location, who can access, insurance/costs	“Your results are confidential. We’ll explain storage, access, and coverage.”
Pediatrics/minors policy	When to test children; consent/assent; childhood-onset vs. adult-onset	“Children are tested only if it affects care now, unless there’s a strong reason.”
After testing—if P/LP	No change for VUS; phenotype-driven care for negative; re-test options	“If uncertain or negative, we follow standard care and watch for updates.”
Recontact & support	How/when recontact happens; portal use; psychosocial resources	“We’ll contact you if things change or new options appear; support is available.”

EHR: electronic health record, P/LP: pathogenic/likely pathogenic, VUS: variant of uncertain significance.

## Data Availability

The data are contained within the article.

## Abbreviations

The following abbreviations are used in this manuscript:

ACC	adrenocortical carcinoma
AML	angiomyolipoma
BAP1-TPDS	BAP1 tumor predisposition syndrome
BHD	Birt-Hogg-Dubé
CNV	Copy-number variant
dMMR	deficient mismatch repair
EAU	European Association of Urology
EMR	electronic medical record
GCT	germ cell tumor
HLRCC	Hereditary Leiomyomatosis and Renal Cell Carcinoma
HPRC	Hereditary Papillary Renal Cell Carcinoma
KS	Klinefelter syndrome
LAM	lymphangioleiomyomatosis
LFS	Li-Fraumeni syndrome
MEN2	Multiple Endocrine Neoplasia type 2
MIBG	metaiodobenzylguanidine
MMR	mismatch repair
NGS	next-generation sequencing
mCRPC	metastatic castration-resistant prostate cancer
MSI	microsatellite instability
OS	overall survival
PD-1	programmed cell death-1
PPGL	pheochromocytoma and paraganglioma
PRRT	peptide receptor radionuclide therapy
PSA	prostate-specific antigen
RCC	renal cell carcinoma
rPFS	radiographic progression-free survival
SSTR	somatostatin receptor
TGCTs	testicular germ cell tumors
UC	urothelial carcinoma
UTUC	upper tract urothelial carcinoma
WES	whole-exome sequencing
WGS	whole-genome sequencing
VHL	von Hippel–Lindau
